# Histone Deacetylase Inhibitors Improve Memory Function and Decrease Neuropathology in APP/PS1 Mice

**DOI:** 10.21203/rs.3.rs-10816126/v1

**Published:** 2026-08-26

**Authors:** Bryan McClarty, Raisa Monteiro, Hongxin Dong

**Affiliations:** Northwestern University; Northwestern University; Northwestern University

**Keywords:** Histone deacetylase (HDAC) inhibitors, H3K9 acetylation, synaptic gene expression, memory, aging, APP/PS1 mice

## Abstract

Although histone deacetylase (HDAC) inhibitors have shown therapeutic potential in aging and Alzheimer’s disease (AD), direct comparisons of nonselective and selective HDAC inhibitors across aging and AD models are lacking. We compared the effects of the broad-spectrum HDAC inhibitor valproic acid (VPA) and the selective class I HDAC inhibitors entinostat (MS-275) and tacedinaline (CI-994) in 3-, 12-, and 18-month-old wild-type and APP/PS1 mice. Mice received 30 days of treatment, followed by a series of behavioral tests assessing different memory domains. Brain tissue was then analyzed for molecular and pathological changes. Both MS-275 and CI-994 improved recognition memory and short-term working memory in 12- and 18-month-old APP/PS1 mice, whereas only CI-994 restored long-term spatial reference memory. CI-994 increased hippocampal synapse-related gene expression and H3K9 acetylation at their promoters, consistent with improved hippocampus-dependent memory. In contrast, MS-275 enhanced synapse-related gene expression and H3K9 acetylation, in the prefrontal cortex (PFC), corresponding to improvements in recognition and working memory. MS-275 also significantly reduced amyloid plaque burden in the hippocampus and PFC, whereas both MS-275 and CI-994 decreased microglial density in APP/PS1 mice. These findings demonstrate distinct spatial and functional effects of selective HDAC inhibition in AD. CI-994 primarily targets hippocampal synaptic plasticity to improve long-term spatial memory, whereas MS-275 preferentially modulates PFC synaptic function and reduces amyloid pathology, leading to improved recognition and working memory. Together, these results identify region-specific HDAC mechanisms that differentially regulate cognitive function and AD pathology, supporting selective HDAC inhibition as a promising therapeutic strategy for AD.

## INTRODUCTION

Class I histone deacetylases (HDACs) regulate a wide range of biological processes, including chromatin remodeling, gene transcription, synaptic plasticity, cell survival, and neuroinflammation [1, 2]. Increasing evidence suggests that dysregulation of HDAC activity contributes to brain aging and the pathogenesis of Alzheimer’s disease (AD). However, the individual contributions of specific class I HDAC isoforms to age-related cognitive decline and AD progression remain incompletely understood.

Pharmacological inhibition of HDACs has emerged as an important strategy for investigating the role of epigenetic regulation in aging and AD. Previous studies have demonstrated that both class I and class II HDAC inhibitors improve memory function in aged rodents [3–7] and restore memory while attenuating AD-related pathologies in transgenic mouse models [8–13]. These beneficial effects are thought to result from enhanced histone acetylation, increased expression of synaptic plasticity-related genes, and suppression of neuroinflammatory pathways. Despite these encouraging findings, it remains unclear whether broad-spectrum and selective HDAC inhibitors exert distinct therapeutic effects on different memory domains and AD-related neuropathology. No study has systematically compared broad-spectrum and selective class I HDAC inhibitors across multiple stages of aging and AD progression to determine their temporal- and region-specific mechanisms of action.

Valproic acid (VPA), a broad-spectrum HDAC inhibitor with established clinical use as an antiepileptic drug, inhibits multiple class I and class II HDACs. Previous studies have reported that VPA improves memory performance and reduces AD-associated pathology in transgenic mouse models [9, 14–19]. However, most of these studies were conducted in relatively young animals and often employed high-dose treatment paradigms, limiting their translational relevance to late-stage disease and clinically relevant dosing. In contrast, entinostat (MS-275), a selective class I HDAC inhibitor that primarily targets HDAC1 and HDAC3, has demonstrated beneficial effects in several neurological disorders by reducing neuroinflammation, gliosis, and amyloid pathology [20–23]. Tacedinaline (CI-994), another selective class I HDAC inhibitor with greater activity toward HDAC1 and HDAC2, has been shown to improve learning and memory during normal aging [7, 24, 25] and exhibits therapeutic potential in several disease models [26–28]. However, whether CI-994 can rescue memory deficits in an AD model has not been investigated.

In this study, we compared the effects of chronic treatment with the broad-spectrum HDAC inhibitor VPA and the selective class I HDAC inhibitors MS-275 and CI-994 in wild-type and APP/PS1 mice at 3, 12, and 18 months of age. We examined multiple memory domains together with synapse-related gene expression, histone H3 lysine 9 acetylation (H3K9ac), amyloid pathology, and microgliosis in the hippocampus and prefrontal cortex (PFC). We hypothesized that selective inhibition of distinct class I HDACs would differentially regulate region-specific epigenetic and transcriptional programs, resulting in unique effects on memory and AD-related pathology. Our findings demonstrate that HDAC1/2- and HDAC1/3-selective inhibition differentially modulate hippocampal and prefrontal cortical synaptic gene expression through H3K9 acetylation and produce distinct improvements in memory function and neuropathology. These results provide new mechanistic insight into the spatial and temporal roles of class I HDACs in aging and AD and support the potential of isoform-selective HDAC inhibitors as precision epigenetic therapies for AD.

## MATERIALS AND METHODS

### Animals

Double-transgenic APP/PS1 mice expressing the chimeric mouse/human APP695 Swedish mutation (APP695swe) and human presenilin-1 exon 9 deletion mutation (PS1-dE9) were used. Male APP/PS1 mice (The Jackson Laboratory, Bar Harbor, ME, USA) were bred with female C57BL/6J mice to generate APP/PS1 and wild-type (WT) littermates. Mice were group-housed (3–5/cage) from weaning until 3, 12, or 18 months of age under a 12-h light/dark cycle with ad libitum access to food and water. All procedures followed the NIH *Guide for the Care and Use of Laboratory Animals* and were approved by the Northwestern University IACUC.

### Drugs

Valproic acid was purchased from Sigma-Aldrich. Entinostat (MS-275; IC_50_ = 0.18 and 0.74 μM for recombinant HDAC1 and HDAC3, respectively, was obtained from Bio-Techne, and tacedinaline (CI-994; IC_50_ = 0.9, 0.9, and 1.4 μM for recombinant HDAC1, HDAC2, and HDAC3, respectively, from MedChemExpress.

The three HDAC inhibitors were dissolved in 2% DMSO. For MS-275 and CI-994, 33.3% PEG-300 was added, and the solutions were brought to the final volume with 0.9% saline and adjusted to pH 6.0–7.0. The doses (VPA: 30mg/kg; MS-275: 10mg/kg; and CI-994: 20mg/kg) were selected based on published studies and our preliminary data [7, 16, 29, 30]. Compounds and vehicle controls were administered intraperitoneally once daily for 30 consecutive days at 10 μL/g body weight.

Mice were monitored daily for general health, respiratory distress, locomotor activity, and body weight. Respiratory distress was defined as persistent rapid, shallow, or labored breathing, and locomotor impairment as markedly reduced spontaneous movement lasting > 30 min after injection. A body-weight loss > 2 g within 2–3 days was considered a treatment-related adverse effect.

### Behavioral Tests

Mice were acclimated to a sound-attenuated testing room for 30 min. Behavioral tests were performed during the light phase in the following order: novel object recognition, Y-maze, and Morris water maze. Testing and data analysis were conducted by an investigator blinded to genotype, age, and treatment.

### Novel Object Recognition (NOR)

Recognition memory was assessed in a Plexiglas arena (40 × 40 × 40 cm) using objects differing in shape, color, and texture [31]. Mice were habituated to the empty arena for 10 min/day for 3 consecutive days. During the first session, locomotor activity and anxiety-like behavior were assessed using the open-field test.

The NOR task consisted of a 10-min acquisition trial, a 1-h intertrial interval, and a 10-min retention trial. During acquisition, mice explored two identical objects. During retention, one familiar object was replaced with a novel object in a counterbalanced manner. Exploration, scored by a blinded experimenter, was defined as touching, leaning on, or sniffing an object within < 1 cm; climbing was excluded. The arena and objects were cleaned with 70% ethanol between trials.

Recognition memory was quantified using the discrimination index (DI):

DI=(Timeexploringthenovelobject−Timeexploringthefamiliarobject)/Totalexplorationtime

Higher DI values indicate better recognition memory.

### Spontaneous Alternation (Y-maze)

Spatial working memory was assessed using a Y-maze with three identical arms (5 × 21 × 15.5 cm) marked with distinct visual cues [32]. Mice were allowed to explore freely for 5 min, and movements were recorded using the Any-Maze tracking system. A spontaneous alternation was defined as consecutive entries into all three arms without revisiting either of the two previously entered arms.


Spontaneousalternation(%)=(Numberofsuccessfulalternations/Totalarmentries−2)×100

### Morris Water Maze (MWM)

Spatial learning and long-term reference memory were assessed using a modified MWM protocol [33]. The maze consisted of a circular pool filled with opaque water maintained at 23°C, containing a hidden escape platform and automated video tracking. Mice were acclimated to the testing room for 30 min before testing.

Training consisted of five consecutive days with four trials/day separated by 1-h intervals. On Day 1, mice were trained with a visible platform, followed by a submerged platform. During acquisition (Days 2–5), the hidden platform remained in a fixed location, and mice were released from randomized starting positions. Spatial cues around the room served as references. Mice had up to 90 s to locate the platform, and escape latency and swim distance were recorded. On Day 6, a 60-s probe trial was conducted with the platform removed. Time and swim distance in the target quadrant were recorded as measures of long-term spatial reference memory.

### Tissue Preparation

Following behavioral testing, mice were transcardially perfused with 0.1 M PBS. Brains were removed and divided into halves on ice. The hippocampus and prefrontal cortex (PFC) from one hemisphere were isolated, snap-frozen, and stored at − 80°C for molecular analyses. The remaining half was fixed overnight in 4% paraformaldehyde, cryoprotected in 30% sucrose, embedded in OCT, and stored for immunohistochemical analysis.

### Chromatin Immunoprecipitation (ChIP)

ChIP was performed using the Magna ChIP^™^ G Tissue Kit (Millipore) according to the manufacturer’s instructions. Tissue was homogenized in lysis buffer with protease inhibitors, and chromatin was sheared by sonication using three 10-s pulses at 75% amplitude on ice. Fragmented chromatin was immunoprecipitated overnight at 4°C with 5 μg anti-H3K9ac antibody and Protein G magnetic beads. DNA-protein complexes were eluted, reverse cross-linked at 65°C for 2 h, digested with proteinase K, precipitated with ethanol, and resuspended in PCR-grade water.

### Quantitative Real-Time PCR

Total RNA from the hippocampus and PFC was extracted using Qiagen kits and reverse-transcribed using the qScript cDNA Synthesis Kit. qPCR was performed using SYBR Green Master Mix on a QuantStudio^™^ 6 Flex system. Primer sequences are listed in Supplementary Table 1. For ChIP-qPCR, input and immunoprecipitated DNA were analyzed in triplicate. Gene expression and ChIP enrichment were quantified using the 2 − ΔΔCt method, with β-actin as the endogenous reference.

### Immunohistochemistry

Brain sections were washed, blocked for 2 h in 10% normal donkey serum, and incubated overnight at 4°C with antibodies against amyloid-β (6E10; 1:250) or Iba1 (1:500). After washing, sections were incubated with Alexa Fluor^®^ 488- or Cy3-conjugated secondary antibodies for 2 h and mounted with ProLong^™^ Glass Antifade Mountant. Images were acquired using a Nikon Eclipse 80i fluorescence microscope. Quantitative analysis was performed using ImageJ. ROIs and fluorescence thresholds were standardized across sections. Protein expression was quantified as immunoreactive area or cell counts, with three sections per brain region averaged for each animal. Negative controls lacking primary or secondary antibodies were included to assess nonspecific staining.

### Statistical Analysis

Data are presented as mean ± SEM. Behavioral and molecular outcomes were analyzed using MANOVA to assess the effects of age, genotype, treatment, and their interactions, followed by Tukey’s post hoc test where appropriate. Neuropathological outcomes, including amyloid plaque burden and microglial density, were analyzed using two-way ANOVA with age and treatment as independent variables, followed by Tukey’s multiple-comparison test. Statistical analyses were performed using IBM SPSS Statistics or GraphPad Prism, and *P* < 0.05 was considered statistically significant.

## RESULTS

### 1. HDAC Inhibitors Improve Memory Function in APP/PS1 Mice

To evaluate the effects of HDAC inhibitors on cognition, recognition memory, short-term working memory, and long-term spatial memory were assessed using novel object recognition NOR, the Y-maze, and the MWM, respectively.

**Figure 1:**
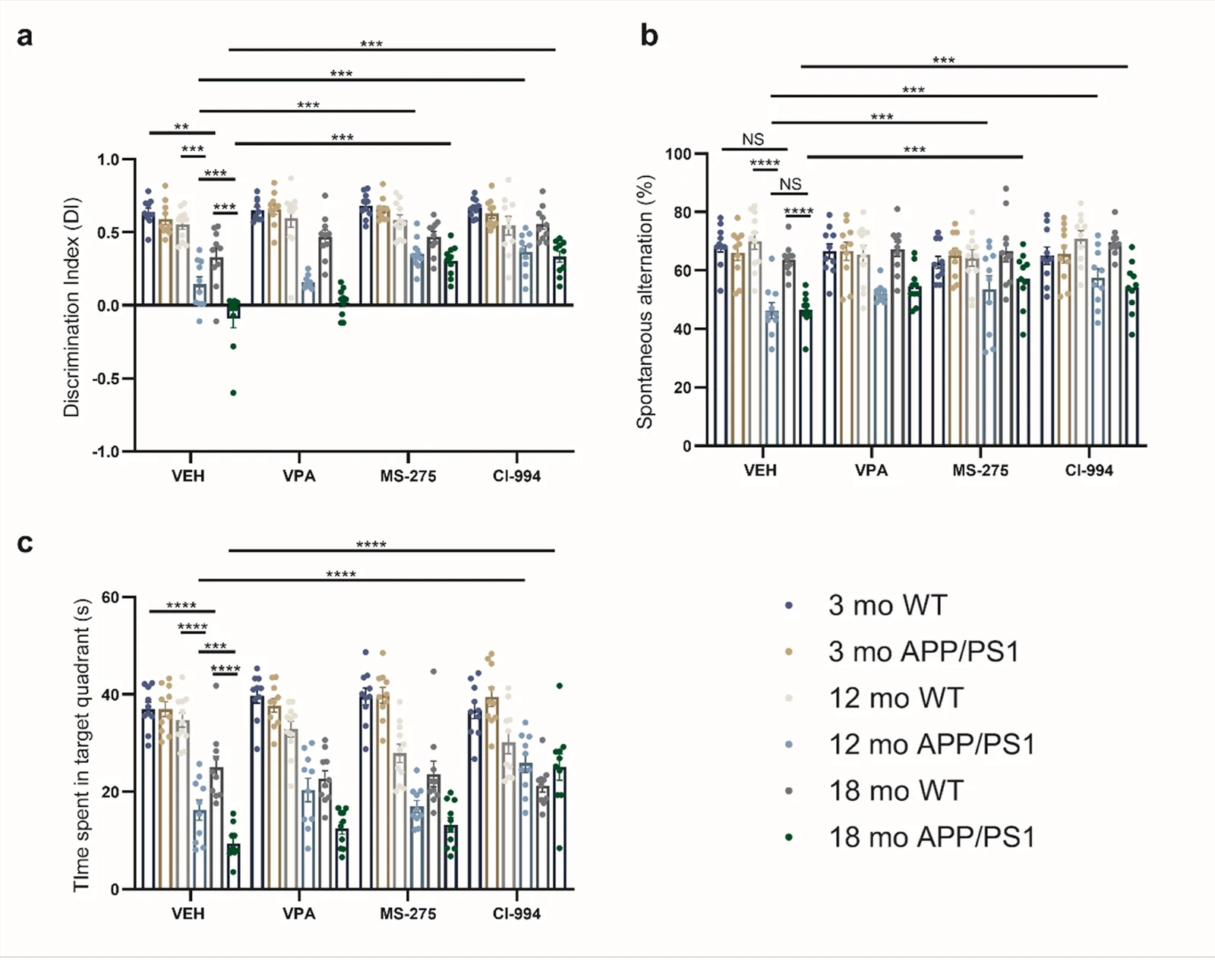
Impact of HDAC inhibitors on different memory domains in APP/PS1 mice. MS-275 and CI-994 significantly improved recognition memory measured by NOR (**a**) and short-term working memory measured by Y-maze (**b**) in both 12- and 18-month-old APP/PS1 mice. Additionally, only CI-994 significantly improved long-term spatial reference memory measured by MWM (**c**) in both 12- and 18-month-old APP/PS1 mice. Data represent mean ± SEM (n = 9–12/group). Blue circles represent 3 mo WT mice, gold circles represent 3 mo APP/PS1 mice, white circles represent 12 mo WT mice, light blue circles represent 12 mo APP/PS1 mice, gray circles represent 18 mo WT mice, green circles represent 18 mo APP/PS1 mice. ***p < 0.001, ****p < 0.0001.

For recognition memory, MANOVA revealed significant main effects of age, genotype, and treatment (all *p* < 0.001), as well as an age × genotype × treatment interaction (*p* = 0.041; Fig. 1a). Aging impaired recognition memory in WT mice, with greater deficits in APP/PS1 mice, particularly at 18 months. MS-275 and CI-994, but not VPA, significantly increased the discrimination index in 12- and 18-month-old APP/PS1 mice (all *p* < 0.001), with no significant effects in aged WT mice.

For short-term working memory, significant main effects of age, genotype, and treatment (all *p* < 0.001), and an age × genotype × treatment interaction (*p* = 0.049), were observed (Fig. 1b). APP/PS1 mice showed reduced spontaneous alternation compared with age-matched WT mice. MS-275 and CI-994, but not VPA, improved performance in 12- and 18-month-old APP/PS1 mice (all *p* < 0.001), with no effects in aged WT mice.

For long-term spatial memory, significant main effects of age, genotype, and treatment (all *p* < 0.001), and an age × genotype × treatment interaction (*p* = 0.047), were observed (Fig. 1c). APP/PS1 mice exhibited age-dependent impairments, and MS-275 and CI-994, but not VPA, significantly increased time spent in the target quadrant in 12- and 18-month-old APP/PS1 mice (all *p* < 0.05).

Together, these findings demonstrate that selective class I HDAC inhibitors, particularly MS-275 and CI-994, improve multiple memory domains in APP/PS1 mice.

### 2. HDAC Inhibitors Rescue Synapse-Related Gene Expression in the Hippocampus and PFC of APP/PS1 Mice

**Figure 2:**
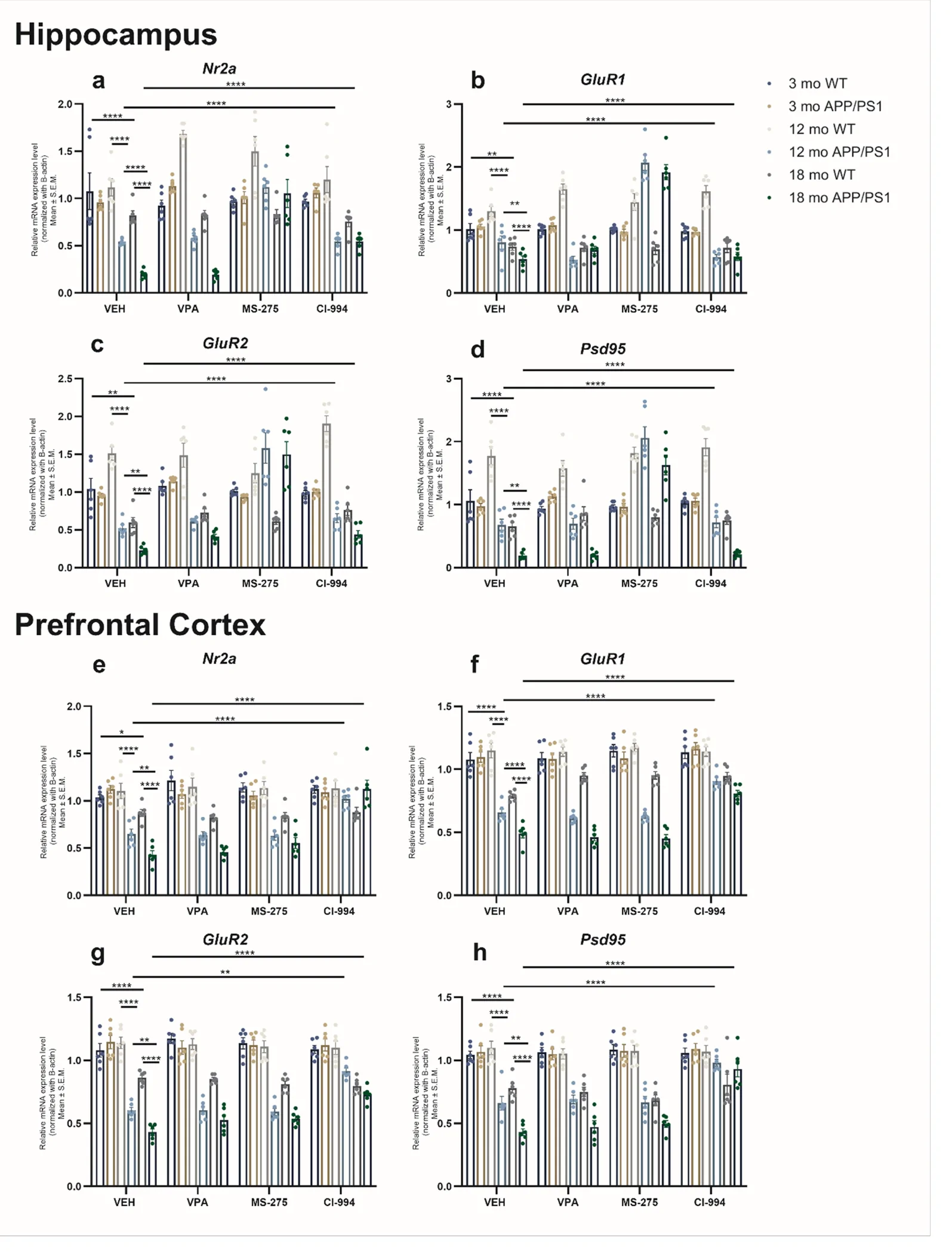
Impact of HDAC inhibitors on synapse-related gene expression in the hippocampus and PFC of APP/PS1 mice. In the hippocampus, mRNA expression of *NR2A* (**a**), *GluR1* (**b**), *GluR2* (**c**), and *PSD95* (**d**) was significantly downregulated with normal aging and was further reduced in aged APP/PS1 mice. Only CI-994 significantly rescued *NR2A, GluR1, GluR2*, and *PSD95* expression in 12- and 18-month-old APP/PS1 mice. In the PFC, *NR2A* (**e**), *GluR1* (**f**), *GluR2*(**g**), and *PSD95* (**h**) expression was significantly downregulated with normal aging and was further reduced in aged APP/PS1 mice. Only MS-275 significantly rescued *NR2A, GluR1, GluR2*, and *PSD95*expression in 12- and 18-month-old APP/PS1 mice. Data represent mean ± SEM (n = 6/group). Blue circles represent 3-month-old WT mice, gold circles represent 3-month-old APP/PS1 mice, white circles represent 12-month-old WT mice, light blue circles represent 12-month-old APP/PS1 mice, gray circles represent 18-month-old WT mice, and green circles represent 18-month-old APP/PS1 mice. ****p < 0.0001.

For *Nr2a, GluR1, GluR2*, and *PSD95* expression in the hippocampus, significant main effects of age, genotype, and treatment and significant age × genotype × treatment interactions were observed (all *p* < 0.05; Fig. 2). Aging reduced expression of all four genes in WT mice, with greater reductions in older APP/PS1 mice. CI-994, but not VPA or MS-275, significantly restored expression of all four genes in 12- and 18-month-old APP/PS1 mice (all *p* < 0.001).

In the PFC, significant effects of age, genotype, treatment, and age × genotype × treatment interactions were observed for all four genes (all *p* < 0.001; Fig. 2). Aging and APP/PS1 genotype reduced synaptic gene expression, particularly in older mice. In contrast to the hippocampus, MS-275, but not VPA or CI-994, significantly increased expression of all four genes in 12- and 18-month-old APP/PS1 mice (all *p* < 0.001). These findings demonstrate region-specific effects, with CI-994 preferentially restoring hippocampal and MS-275 PFC synaptic gene expression.

### 3. HDAC Inhibitors Restore H3K9 Acetylation at Synaptic Gene Promoters

**Figure 3:**
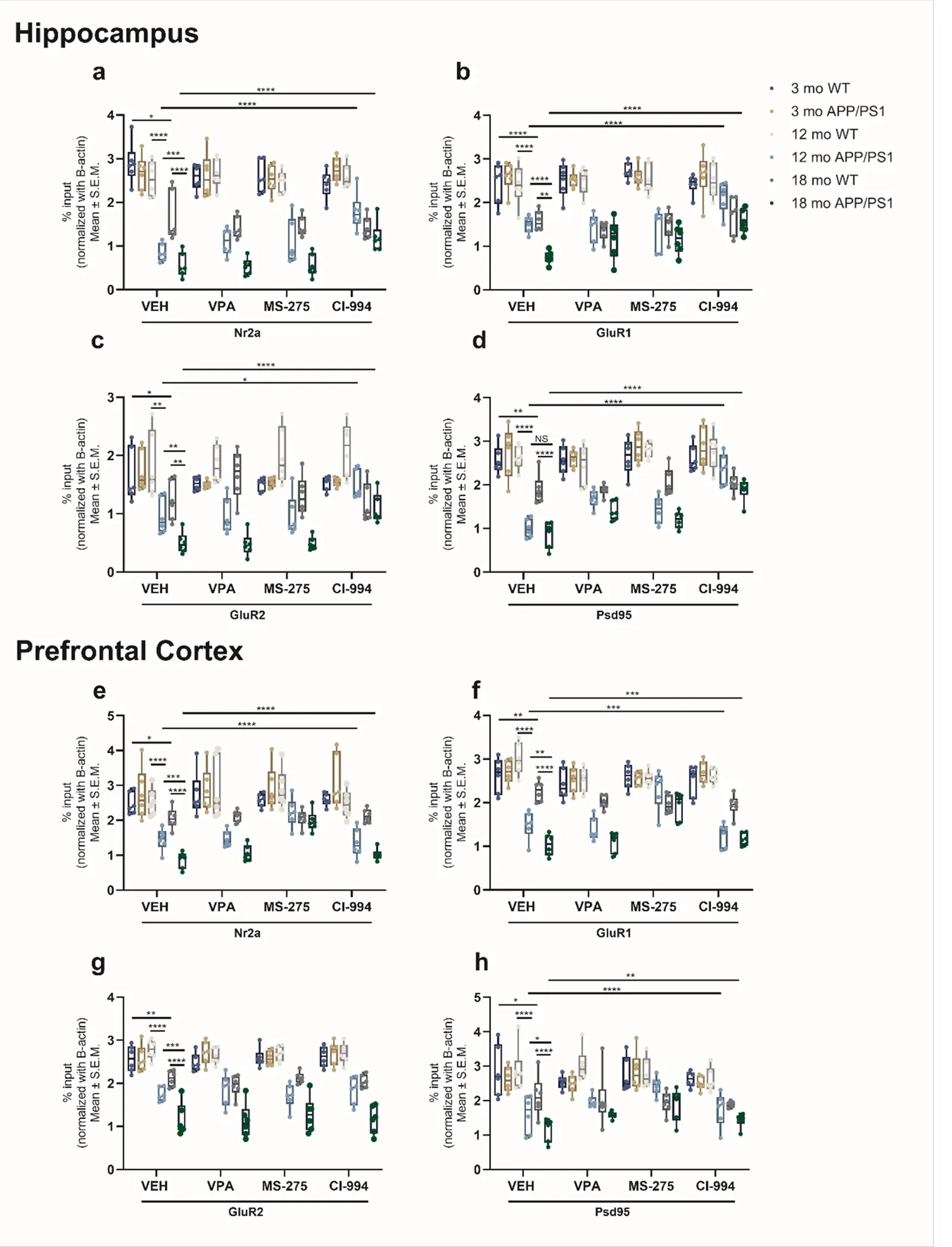
Impact of HDAC inhibitors on H3K9ac levels at synaptic gene promoters in the hippocampus and PFC of APP/PS1 mice. In the hippocampus, H3K9ac levels at synapse-related gene promoters decreased with normal aging and were further reduced in aged APP/PS1 mice. Only CI-994 significantly rescued H3K9ac levels at the *Nr2a* (**a**), *GluR1* (**b**), *GluR2*(**c**), and *PSD95* (**d**) promoters in 12- and 18-month-old APP/PS1 mice. In the PFC, H3K9ac levels at synapse-related gene promoters decreased with normal aging and were further reduced in aged APP/PS1 mice. Only MS-275 significantly rescued H3K9ac levels at the *Nr2a* (**e**), *GluR1*(**g**), and *PSD95* (**h**) promoters, but not the *GluR2* (**f**) promoter, in 12- and 18-month-old APP/PS1 mice. Data represent mean ± SEM (n = 6/group). Blue circles represent 3-month-old WT mice, gold circles represent 3-month-old APP/PS1 mice, white circles represent 12-month-old WT mice, light blue circles represent 12-month-old APP/PS1 mice, gray circles represent 18-month-old WT mice, and green circles represent 18-month-old APP/PS1 mice. *p < 0.05, ***p < 0.001, ****p < 0.0001.

We next examined H3K9 acetylation (H3K9ac) at the promoters of *Nr2a, GluR1, GluR2*, and *PSD95* in the hippocampus and PFC. In the hippocampus, significant effects of age, genotype, treatment, and age × genotype × treatment interactions were observed (all *p* < 0.05; Fig. 3). Aging reduced H3K9ac at all four promoters, with further reductions in APP/PS1 mice. CI-994, but not VPA or MS275, significantly increased H3K9ac enrichment at all four promoters in 12- and 18-month-old APP/PS1 mice (all *p* < 0.05).

In the PFC, aging and APP/PS1 genotype also reduced H3K9ac at synaptic gene promoters. Consistent with the gene expression findings, MS-275, but not VPA or CI-994, restored H3K9ac at the *Nr2a, GluR1*, and *PSD95* promoters (all *p* < 0.001), whereas *GluR2* H3K9ac was unaffected by treatment. Thus, the region-specific restoration of synaptic gene expression was accompanied by corresponding increases in promoter-associated H3K9ac, supporting an epigenetic mechanism.

### 4. MS-275, but Not VPA or CI-994, Attenuates Amyloid Pathology

**Figure 4:**
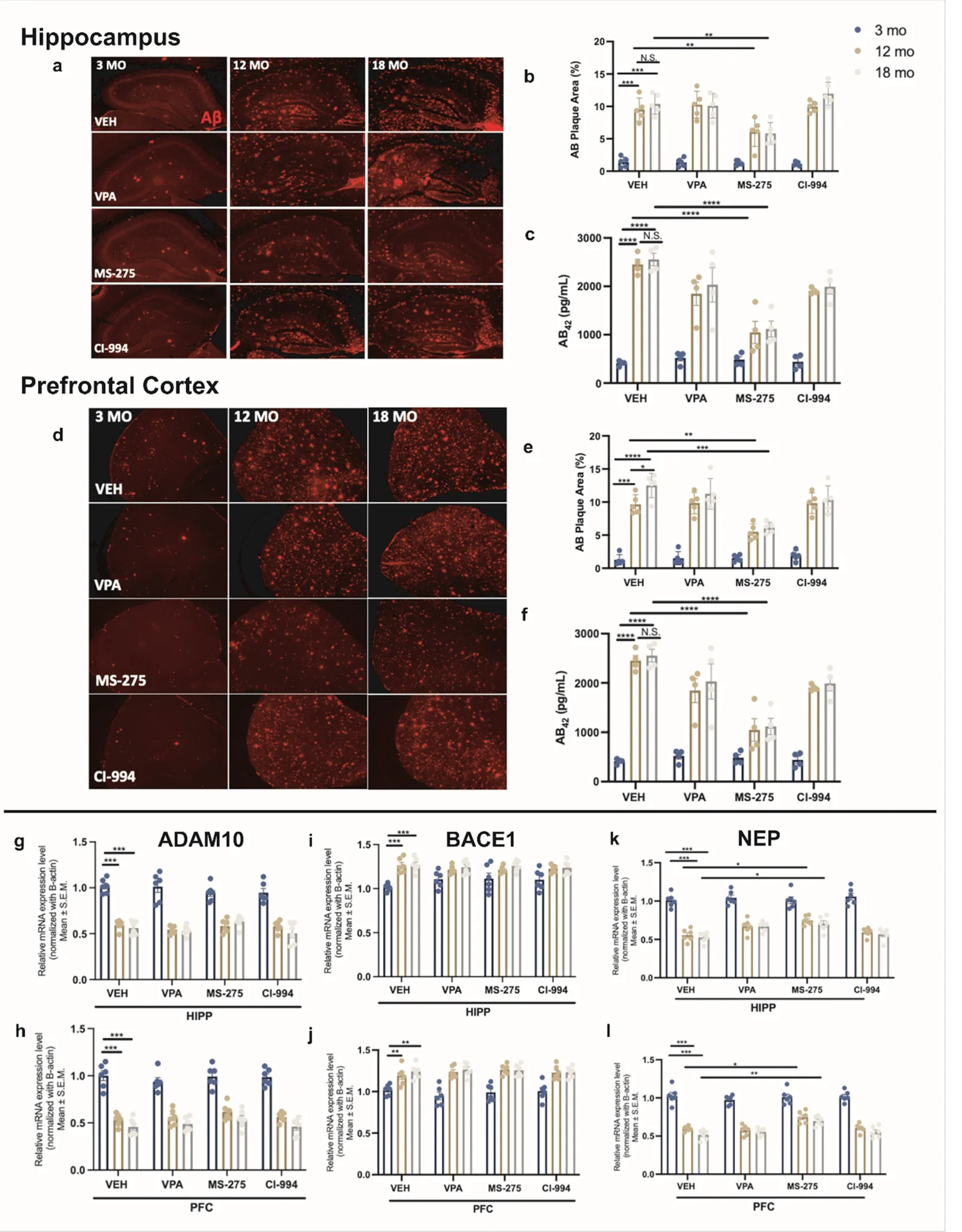
Impact of HDAC inhibitors on amyloidosis and downstream pathways in the hippocampus and PFC of APP/PS1 mice. Aβ immunostaining in the hippocampus (**a**) and PFC (**d**), quantification of Aβ plaques in the hippocampus (**b**) and PFC (**e**), and Aβ42 ELISA in the hippocampus (**c**) and PFC (**f**). Chronic MS-275 treatment significantly reduced Aβ plaque burden and Aβ42 levels in both the hippocampus and PFC. mRNA expression of ADAM10 in the hippocampus (**g**) and PFC (**h**), BACE1 in the hippocampus (**i**) and PFC (**j**), and NEP in the hippocampus (**k**) and PFC (**l**) was also assessed. The three HDAC inhibitors had no significant effect on ADAM10 or BACE1 expression, whereas MS-275 significantly increased NEP expression. Data represent mean ± SEM (n = 6/group). Blue circles represent 3-month-old APP/PS1 mice, gold circles represent 12-month-old APP/PS1 mice, and white circles represent 18-month-old APP/PS1 mice. ***p < 0.001.

To determine whether HDAC inhibitors alter amyloid pathology, we quantified Aβ plaque burden and Aβ42 levels in the hippocampus and PFC of APP/PS1 mice. Both measures increased significantly with age in both brain regions, with significant effects of age, treatment, and age × treatment interactions (all *p* ≤ 0.001; Fig. 4a-f). MS-275, but not VPA or CI-994, significantly reduced Aβ plaque burden and Aβ42 levels in 12- and 18-month-old APP/PS1 mice (all *p* < 0.05).

To investigate the underlying mechanism, we examined genes involved in APP processing and Aβ degradation. ADAM10 and BACE1 showed age-related changes but were unaffected by treatment, suggesting that HDAC inhibitors did not alter APP processing. In contrast, MS-275, but not VPA or CI-994, significantly increased expression of the Aβ-degrading enzyme NEP in both brain regions of 12- and 18-month-old APP/PS1 mice (all *p* < 0.05; Fig. 4k, l). These findings suggest that the anti-amyloid effects of MS-275 may involve enhanced Aβ clearance through increased NEP expression.

### 5. MS-275 and CI-994 Reduce Microglial Activation During AD Progression

**Figure 5:**
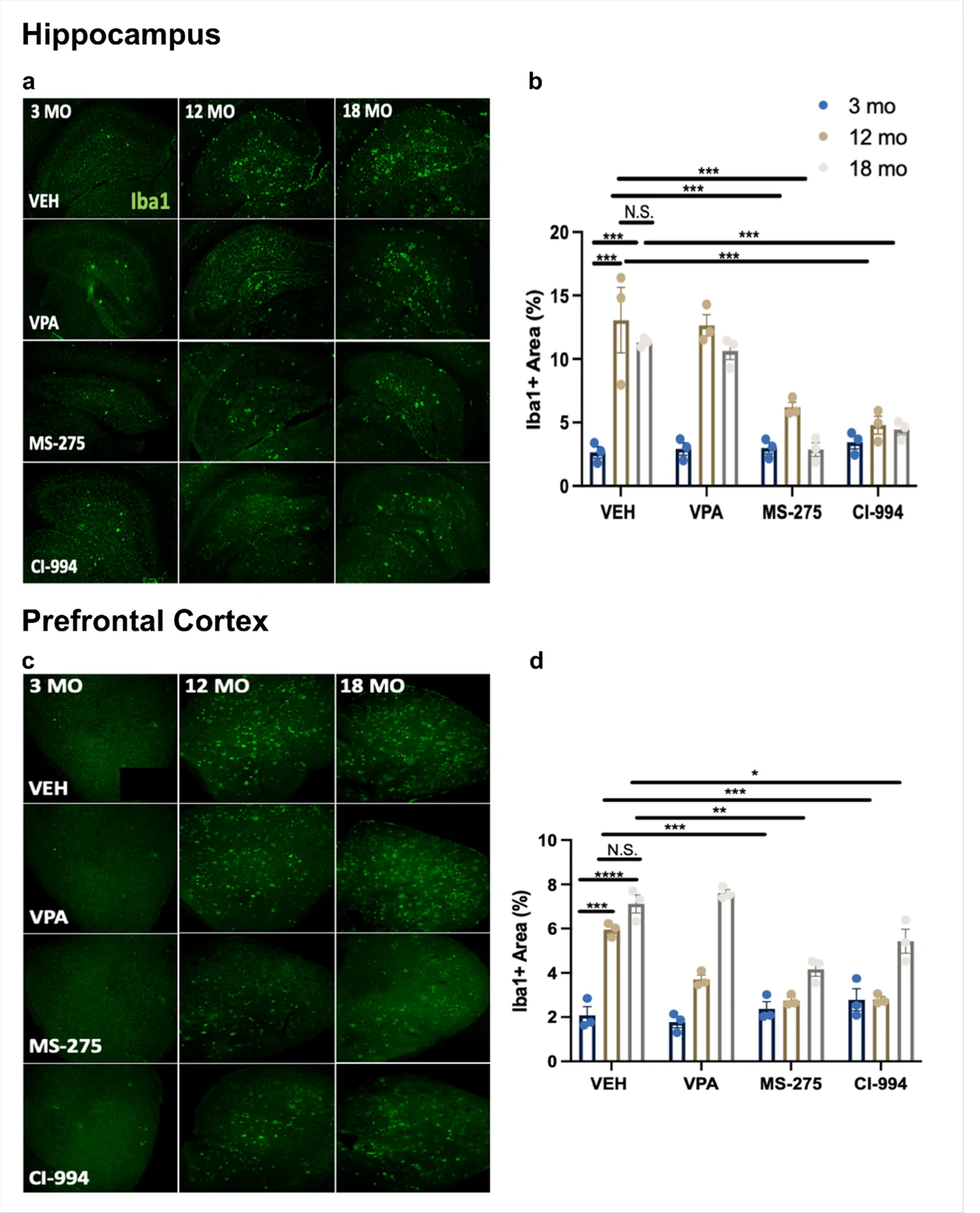
Impact of HDAC inhibitors on microglial cell density in the hippocampus and PFC of APP/PS1 mice. Microglial cell density was quantified using Iba1+ immunostaining in the hippocampus (**a, b**) and PFC (**c, d**). Chronic treatment with MS-275 and CI-994 significantly decreased Iba1+ cell density. Data represent mean ± SEM (n = 6/group). Blue circles represent 3-month-old APP/PS1 mice, gold circles represent 12-month-old APP/PS1 mice, and white circles represent 18-month-old APP/PS1 mice. ***p < 0.001.

To assess neuroinflammation, Iba1-positive (Iba1^+^) microglial density was quantified in the hippocampus and PFC. Significant effects of age, treatment, and age × treatment interactions were observed in both regions (all *p* < 0.0001; Fig. 5). Iba1^+^ microglial density increased significantly in 12- and 18-month-old APP/PS1 mice. Chronic treatment with MS-275 and CI-994, but not VPA, significantly reduced microglial density in both the hippocampus and PFC (all *p* < 0.05).

Collectively, these findings demonstrate that selective class I HDAC inhibitors exert distinct but complementary therapeutic effects during AD progression. CI-994 preferentially restored hippocampal synaptic gene expression and H3K9ac, whereas MS-275 improved PFC synaptic function, reduced amyloid pathology, increased NEP expression, and attenuated microglial activation. Both compounds improved cognitive function and reduced neuroinflammation, while VPA showed no significant therapeutic benefit.

### 6. Behavioral and Molecular Correlation Analyses

Results and Figures are in Supplementary Section. Collectively, these findings demonstrate that restoration of synaptic gene expression is strongly associated with cognitive improvement following selective class I HDAC inhibition. Moreover, the cognitive benefits of MS-275 are closely associated with reduced cortical amyloid burden, whereas hippocampal spatial memory appears to be more closely linked to synaptic gene restoration than to amyloid plaque load.

## DISCUSSION

In the present study, we used a pharmacological approach with selective class I HDAC inhibitors to define the roles of HDAC1–3 in memory function and AD-related pathology during aging and disease progression. Our findings demonstrate that class I HDACs regulate distinct memory domains and AD pathologies in a region- and stage-specific manner. Specifically, CI-994, which preferentially inhibits HDAC2, improved hippocampus-dependent memory by restoring the expression of key synaptic genes (*Nr2a, GluR1, GluR2*, and *PSD95*) together with increased H3K9 acetylation at their promoters. In contrast, MS-275, which preferentially inhibits HDAC1/3, improved prefrontal cortex-dependent recognition and working memory while restoring synaptic gene expression and increasing H3K9 acetylation, particularly at the *Nr2a and PSD95* promoters. Notably, neither inhibitor significantly altered memory performance in aged wild-type mice, suggesting that selective class I HDAC inhibition primarily restores pathological epigenetic dysregulation rather than enhancing normal memory function. In addition to improving memory, MS-275 reduced amyloid plaque burden and Aβ42 levels in both the hippocampus and PFC, whereas both MS-275 and CI-994 attenuated microgliosis in aged APP/PS1 mice. Surprisingly, at the selected dose, we did not observe any effects of VPA on either memory performance or AD-related pathology in the AD mouse model at any age. These negative findings may be attributable to the low dose rate utilized and the relatively low binding affinity and weak overall potency of VPA toward Class I HDACs in vivo [34, 35] when compared with the high-potency, isoform-selective profiles of MS-275 and CI-994 [36–38]. Together, these findings support a model in which distinct class I HDAC isoforms regulate different molecular pathways underlying cognitive decline and AD pathology in a spatially and temporally specific manner.

Our data demonstrate that CI-994 was the only inhibitor that robustly restored synaptic gene expression and H3K9 acetylation at multiple synaptic gene promoters in the hippocampus, leading to significant improvements in hippocampus-dependent memory. These findings are consistent with our previous observations that HDAC2 is selectively enriched at synaptic gene promoters during aging and AD, where it represses transcription of genes required for synaptic plasticity [39]. Although CI-994 inhibits both HDAC1 and HDAC2, the concordance between our previous chromatin studies and the present pharmacological data strongly suggests that HDAC2 is the principal regulator of hippocampal synaptic plasticity and long-term memory. Our findings are also consistent with previous studies showing that elevated HDAC2 expression contributes to age-related cognitive decline and AD by suppressing genes involved in learning and memory [24, 40–42]. Our work further establishes HDAC2 as a key epigenetic regulator of hippocampal memory and support HDAC2 inhibition as a potential therapeutic strategy for cognitive impairment in AD.

In contrast, MS-275 selectively restored synaptic gene expression and promoter H3K9 acetylation in the PFC and significantly improved recognition and short-term working memory. These findings are consistent with our previous evidence demonstrating increased HDAC3 occupancy at synaptic gene promoters in the PFC during AD progression [39]. Although MS-275 inhibits both HDAC1 and HDAC3, accumulating evidence indicates that HDAC3 plays a particularly important role in cortical synaptic plasticity and cognitive flexibility. Specifically, both the genetic deletion and pharmacological inhibition of HDAC3 have been demonstrated to enhance synaptic plasticity and long-term memory formation, such as object recognition and spatial location memory [43–47]. Our findings demonstrate that HDAC3-dependent epigenetic repression contributes to synaptic dysfunction specifically within the PFC, suggesting that HDAC3 may preferentially regulate cortical memory processes rather than hippocampal-dependent memory.

An unexpected and important finding was that MS-275, but not CI-994, significantly reduced amyloid plaque burden and Aβ42 levels in both the hippocampus and PFC. Interestingly, these reductions were accompanied by increased expression of NEP, one of the major Aβ-degrading enzymes, whereas expression of APP-processing enzymes, including ADAM10 and BACE1, was unchanged. These results suggest that the reduction in amyloid pathology is unlikely to result from altered APP processing, but rather from enhanced Aβ clearance. Previous studies have shown that HDAC1 and HDAC3 repress NEP transcription through histone deacetylation at the NEP promoter [48, 49]. Thus, inhibition of HDAC1 and HDAC3 by MS-275 may increase histone acetylation at the NEP promoter, leading to enhanced NEP expression and accelerated Aβ degradation. Consistent with this mechanism, our behavioral correlation analyses further demonstrated that reduced cortical amyloid burden was strongly associated with improvements in recognition and working memory, suggesting that enhanced Aβ clearance contributes to the cognitive benefits of MS-275.

Neuroinflammation is another major pathological hallmark of AD and contributes to synaptic dysfunction, neuronal loss, and cognitive decline [50–53]. Microglia serve as the resident innate immune cells of the central nervous system and exhibit both protective and detrimental functions depending on their activation state and local microenvironment [54–56]. Using Iba1 immunostaining, we found that both MS-275 and CI-994 significantly reduced microglial density in the hippocampus and PFC of aged APP/PS1 mice, suggesting that class I HDAC inhibition attenuates neuroinflammatory responses during AD progression. These findings agree with previous studies showing that pharmacological inhibition or genetic suppression of class I HDACs reduces microglial activation and inflammatory signaling [20, 57–62].

Overall, our findings support a model in which HDAC2 predominantly regulates hippocampal synaptic plasticity and memory formation, whereas HDAC3 preferentially controls cortical synaptic function and executive cognition. In parallel, HDAC1 and HDAC3 inhibition reduces amyloid pathology through enhanced Aβ clearance and suppresses neuroinflammation by limiting microglial responses. These findings highlight the functional heterogeneity of class I HDACs in the aging brain and suggest that isoform-selective HDAC inhibition represents a promising therapeutic strategy capable of simultaneously targeting epigenetic dysregulation, synaptic dysfunction, amyloid pathology, and neuroinflammation in AD.

Although our findings support the therapeutic potential of selective HDAC inhibitors for AD, several limitations should be acknowledged, and future studies are warranted to further elucidate their mechanisms of action, optimize treatment paradigms, and evaluate their long-term efficacy and safety. First, CI-994 and MS-275 are preferential rather than fully isoform-selective inhibitors. Therefore, although our results, together with prior chromatin occupancy data, support distinct roles for HDAC2 in the hippocampus and HDAC3 in the PFC, contributions from HDAC1 and potential off-target effects cannot be excluded. Future studies using region- and cell type-specific genetic manipulation of individual HDAC isoforms will be important to establish causal mechanisms. Second, the present study examined a limited set of synaptic genes and histone modifications. Although changes in H3K9 acetylation at synaptic gene promoters were associated with restored gene expression and improved memory, broader epigenomic and transcriptomic analyses are needed to define the full set of HDAC-regulated pathways underlying the behavioral and pathological effects observed here. In particular, chIP assay at the NEP promoter would help determine whether MS-275 directly increases histone acetylation and transcriptional activity at this locus. Third, the APP/PS1 model recapitulates amyloid pathology but does not fully capture all features of sporadic AD, including robust tau pathology, extensive neuronal loss, and the complex contributions of aging-associated comorbidities. Thus, the therapeutic relevance of selective class I HDAC inhibition should be evaluated in additional AD models and in both sexes across multiple stages of disease progression. Finally, although both inhibitors reduced Iba1-positive microglial density, Iba1 labels both resting and activated microglia, our data cannot distinguish whether HDAC inhibition primarily reduces microglial activation, proliferation, or both. Future studies incorporating activation-specific markers such as CD68, together with homeostatic markers including CD11b, P2RY12, or TMEM119, as well as single-cell transcriptomic analyses, will be important for defining how selective class I HDAC inhibition reshapes microglial phenotypes and neuroimmune signaling during AD progression.

In summary, our findings demonstrate that selective pharmacological inhibition of class I HDACs produces region- and stage-specific benefits in an AD mouse model. CI-994 preferentially restored hippocampal synaptic gene expression and H3K9 acetylation, leading to improved hippocampus-dependent memory, consistent with a central role for HDAC2 in hippocampal synaptic plasticity. In contrast, MS-275 preferentially improved PFC-dependent recognition and working memory, accompanied by restored synaptic gene expression and promoter H3K9 acetylation, supporting a prominent role for HDAC3 in cortical cognitive function. MS-275 also reduced amyloid plaque burden and Aβ42 levels, potentially through increased NEP-mediated Aβ clearance, whereas both inhibitors attenuated microglial responses. These results highlight the functional heterogeneity of class I HDACs and support isoform-selective HDAC inhibition as a promising strategy to target epigenetic dysregulation, synaptic dysfunction, amyloid pathology, and neuroinflammation in AD.

## Supplementary Material

Supplementary Files

This is a list of supplementary files associated with this preprint. Click to download.

• SupplementarySectionHistoneDeacetylaseInhibitorsImproveMemoryFunctionandDecreaseNeuropathologyinAPPPS1Mice.docx

## Data Availability

All data generated from this study will be available upon request or through publicly accessible repositories.
